# Sex Difference in Risk Factors, GRACE Scores, and Management among Post-Acute Coronary Syndrome Patients in Sri Lanka

**DOI:** 10.1155/2020/4560218

**Published:** 2020-07-30

**Authors:** Priyadarshani Galappatthy, Vipula Bataduwaarachchi, Priyanga Ranasinghe, Gamini Galappatthy, Upul Senerath, Chandrika Wijeyaratne, Ruwan Ekanayake

**Affiliations:** ^1^Department of Pharmacology, Faculty of Medicine, University of Colombo, Colombo, Sri Lanka; ^2^Ministry of Health, Nutrition and Indigenous Medicine, Colombo, Sri Lanka; ^3^Department of Community Medicine, Faculty of Medicine, University of Colombo, Colombo, Sri Lanka; ^4^Department of Gynecology and Obstetrics, Faculty of Medicine, University of Colombo, Colombo, Sri Lanka; ^5^Ceylon College of Physicians, Colombo, Sri Lanka; ^6^Sri Lanka Heart Association, Colombo, Sri Lanka

## Abstract

**Objective:**

To assess sex-based differences in the prevalence of risk factor, their management, and differences in the prognosis among acute coronary syndrome (ACS) in Sri Lanka.

**Methods:**

Patients diagnosed with ACS were recruited from hospitals throughout the island. The Joint European Societies guidelines were used to assess recommended targets for coronary heart disease risk factors, and the GRACE score was used to assess the post-ACS prognosis. Age-adjusted regression was performed to calculate odds ratios for men versus women in risk factor control.

**Results:**

A total of 2116 patients, of whom 1242 (58.7%) were men, were included. Significant proportion of women were nonsmokers; OR = 0.11 (95% CI 0.09 to 0.13). The prevalence of hypertension (*p* < 0.001), diabetes (*p* < 0.001), and dyslipidemia (*p*=0.004) was higher in women. The LDL-C target was achieved in a significantly higher percentage of women (12.6%); OR = 0.33 (95% CI 0.10 to 1.05). When stratified by age, no significant differences were observed in achieving the risk factor targets or management strategies used except for fasting blood sugar (*p* < 0.05) where more men achieved control target in both age categories. Majority of the ACS patients had either high or intermediate risk for one-year mortality as per the GRACE score. In-hospital and 1-year mean mortality risk was significantly higher among men of less than 65 years of age (*p* < 0.05).

**Conclusions:**

Smoking is significantly lower among Sri Lankan women diagnosed with ACS. However, hypertension, diabetes, and dyslipidemia were more prevalent among them. There was no difference in primary and secondary preventive strategies and management in both sexes but could be further improved in both groups.

## 1. Introduction

Acute coronary syndrome (ACS) is a major cause of death and disability in the world [1]. Despite new developments in management, many studies conducted on patients with ACS during past two decades have shown that the prevalence and mortality associated with ACS is significantly higher in women than in men [2]. For example, the CURE trials have demonstrated better outcomes with clopidogrel in addition to aspirin among men compared with women with coronary artery disease [3]. It is uncertain whether this sex difference in prognosis is related to different physiological and baseline characteristics. According to some studies, it can be attributed to differences in the diagnostic and therapeutic measures employed. For example, it has been shown that women undergo coronary revascularization and other invasive procedures less frequently than men [4]. The Gulf RACE study reported significant sex differences in the management and outcomes of ACS patients from six Gulf countries [5]. The SURF phase 1 audit showed poor risk factor management among women with coronary heart disease (CHD) in Europe, Asia, and the Middle East, which represent three different ethnically and geographically diverse populations [6, 7].

South Asians, or people who trace their ancestry to the Indian subcontinent, have been observed to exhibit higher indicators of cardiometabolic risk relative to White populations [8]. The region has seen an epidemiological shift from communicable to noncommunicable diseases (NCDs) in the last few decades due to improved socioeconomic conditions and high rates of rural to urban migration [9]. Studies have shown that the South Asian ethnicity is associated with higher incidence of CHD [10]. South Asians are also believed to have an earlier presentation of CHD compared with other ethnicities [11]. However, large scale national data on prevalence, risk factors and management of ACS are generally not available among the South Asian populations, with the exception of few regional studies. Furthermore, sex difference in risk factors and management of ACS have not been explored in detail in this population.

Sri Lanka is a rapidly developing island nation in the South Asian region that has a population of nearly 21 million. The country is presently facing a high burden of NCDs, including cardiovascular diseases, cancers, and diabetes [12]. Studies have shown an increasing trend of premature mortality due to NCDs in Sri Lanka during the past decade and NCDs had been responsible for 71% of all deaths in Sri Lanka in 2014 [12]. The Acute Coronary Syndrome Sri Lanka Audit Project (ACSSLAP) is the first nationwide study to assess ACS in the Sri Lankan population [13]. In the present study, we use the ACSSLAP data to investigate whether there are sex differences in the presentation and the management of CHD risk factors among patients with ACS in Sri Lanka.

## 2. Materials and Methods

### 2.1. Study Population

Details of the study protocol and the methodology of ACSSLAP have been previously published [13]. In summary, during a 4-week window, consecutive patients with ACS were recruited from all secondary and tertiary care hospitals in the country, in all 25 districts belonging to the 9 administrative provinces of Sri Lanka. Data were collected consecutively from each hospital to include a minimum of up to 30 patients diagnosed with ACS from each institution. This number of 30 patients per institution was selected as per the International Network on Rational Use of Drugs (INRUD) recommendation on the number of prescriptions needed per facility for studying drug use indicators. Ethics approval was obtained from the Ethics Review Committee of Faculty of Medicine, University of Colombo. Administrative approvals were obtained from the Director General of Health Services, Provincial Directors, Directors of the respective hospitals of the Ministry of Health, and the consultants of each ward.

### 2.2. Data Collection, Definitions, and Risk Factor Targets

A common case record form (CRF), with standardised criteria, was developed by the investigators. Data collection was done by perusal of medical records and interviewing of patients and junior doctors. The CRF had several sections, which evaluated sociodemographic details (age, sex, and ethnicity), past history of medical conditions, risk factors (e.g., smoking and alcohol consumption), details of current admission (diagnosis, clinical parameters, managements including reperfusion therapy, and results of investigations, including cardiac assessment), and details about plans on discharge for follow-up care. Only the complete data sheets were taken for the analysis. Patients were classified by their primary discharge diagnosis into one of the following three groups: STEMI/left bundle branch block (LBBB), NSTEMI, and unstable angina (UA). Random monitoring of all CRF (2–5%) forms for data accuracy and quality was performed during and in the weeks after enrolment across study settings.

The Joint European Societies guidelines were used to assess recommended targets for CHD risk factors [14, 15]. Obesity was defined as body mass index (BMI) more than 30 kg/m^2^. Blood pressure targets were <140/80 mmHg and <140/90 mmHg for patients with and without diabetes, respectively. Fasting blood sugar (FBS) target for patients with diabetes was <7 mmol/L. The targets for total cholesterol (TC) and low-density lipoprotein (LDL) cholesterol were <3 mmol/L (<55 mg/dL) and <1.8 mmol/L (<32.4 mg/dL), respectively. GRACE score (the global registry of acute coronary events) was used in risk stratification of ACS patients [16]. The web version of the GRACE 2.0 ACS risk calculator was used to calculate the GRACE scores for the individual patients to assess the in-hospital mortality risk and the post-discharge to 6 months, 1 years, and 3 years and mortality risk following ACS (available at http://www.gracescore.org/website/WebVersion.aspx).

### 2.3. Statistical Analysis

Patient characteristics are presented as means (SD) and percentages for continuous and categorical variables, respectively. For normally distributed numeric data, intergroup comparisons were conducted with a Student's *t* test or ANOVA with post hoc analysis, while for nonnormally distributed numeric data, the Mann–Whitney *U* test was used. Age-adjusted logistic regression analysis was used to obtain odds ratios (OR, 95% CI) of men and women for individual risk factor targets and GRACE risk categories. Categorization was done according to age group (<65 years and >65 years) and level of health care delivery. We also assessed any difference of findings between ACS categories (STEMI, NSTEMI, and UA). All analyses were performed with SPSS version 20 (SPSS Inc., Chicago, IL, USA). In all analyses, a *p* value < 0.05 was considered statistically significant.

## 3. Results

A total of 2116 patients with ACS were recruited, of whom 1242 (58.7%) were men. Patient characteristics, cardiovascular risk factors, and the management stratified by sex are summarized in Table 1. On average, at the time of presentation, women were 2 years older than men (*p* < 0.001). STEMI was significantly more common among men (*p* < 0.001), while UA was significantly common among women (*p* < 0.001). Smoking was significantly higher among men, except passive smoking which was commoner among women (*p* < 0.001). A significantly higher percentage of women had hypertension (*p* < 0.001), dyslipidemia (*p*=0.004), and diabetes (*p* < 0.001). LDL-C levels were significantly higher among men (*p*=0.008). With regard to primary preventive medications, aspirin (*p*=0.013), statins (*p*=0.002), and ARB/ACE inhibitor (*p* < 0.001) use were better among women. No significant sex differences were observed in the secondary prevention medications. With regard to the discharge plan, a significantly higher percentage of women received dietary advices *p*=0.017), while angiograms (*p*=0.006) and 2D-Echo (*p*=0.001) were planned more in men.

### 3.1. Risk Factors, GRACE Risk Assessment, and In-Hospital Management

**Figure 1:**
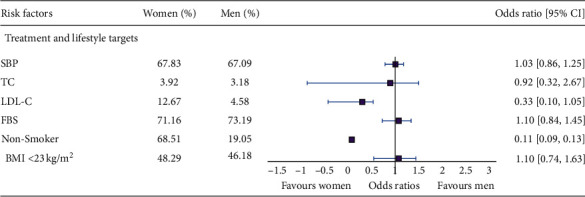
Age-adjusted sex differences in the risk factor management.

Control of cardiovascular risk factors was suboptimal in both men and women for all risk factors observed (Figure 1). SBP target was achieved by an almost equal number (67%) of men and women. A significant proportion of women were nonsmokers; the OR for being a nonsmoker was 0.11 (95% CI 0.09 to 0.13). There was no significant difference between sex in achieving TC, FBS, and BMI targets. LDL-C target was achieved by a significantly higher percentage of women (12.6%); the OR was 0.33 (95% CI 0.10 to 1.05). The average serum creatinine level is significantly higher (<0.001) among men which may indicate the normal difference between genders according to the differences in the muscle mass. The average haemoglobin level is significantly lower among women, and both men (12.6 g/dL) and women (11.5 g/dL) fall in the anemic category as per the WHO definitions (men, 13 g/dL; women, 12 g/dL).

Age-adjusted sex differences on lifestyle factors are shown. Odds ratios (95% CI) are presented as women versus men. SBP, systolic blood pressure; TC, total cholesterol; LDL-C, low-density lipoprotein cholesterol; FBS, fasting blood sugar; BMI, body mass index.

**Figure 2:**
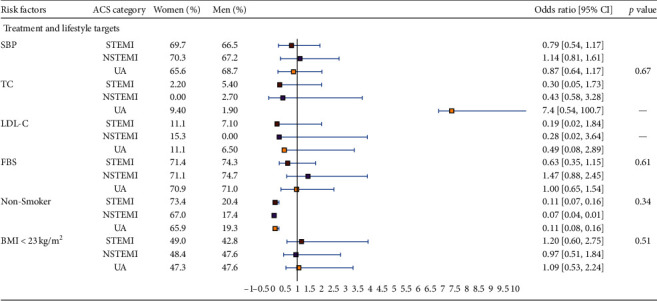
Age-adjusted sex differences in lifestyle factor management, stratified by the types of acute coronary syndrome. Odds ratios (95% CI) are presented as women versus men; *p* values are for interaction between subgroups. STEMI, ST elevation myocardial infarction; NSTEMI, non-ST elevation myocardial infarction; UA, unstable angina; SBP, systolic blood pressure; TC, total cholesterol; LDL-C, low-density lipoprotein cholesterol; FBS, fasting blood sugar; BMI, body mass index.

In the GRACE risk assessment, majority of the ACS patients were either high (women = 41.39%; men = 43.69%) or intermediate (women = 43.32%; men = 41.99%) risk for one-year mortality (Figure 2). GRACE risk assessment of post-ACS patients stratified by sex and the risk levels are presented in Table 2. In-hospital mean mortality risk was significantly higher among men of less than 65 years of age (Table 2) (*p* < 0.05). One-year post-discharge mortality of men less than 65 years was also significantly high (*p* < 0.02). No significant difference observed between men and women in other GRACE risk categories. Men were common among high and intermediate one-year and three-year post-discharge mortality risk categories; however, the differences were not statistically significant.

With regard to the secondary prevention strategies followed at the in-hospital setting, more men received care compared with women for all the items checked (Table 1). Percentages of patients who received dietary advices (78.0%), 2D-Echo (61.8%), and cardiology referral (60.5%) were the highest among all the management items in the total population, although still not meeting the required standards (Table 1).

### 3.2. Sex Difference by ACS Type and Age

The sex difference in achieving treatment targets differed between ACS types. Although there was no significant difference observed between ACS subtypes, achieving TC targets were higher among women with UA (9.4% compared to 1.9%). However, this finding was confounded by a smaller number of observations. Nonsmokers were common among women across all three ACS types (Figure 2).

**Figure 3:**
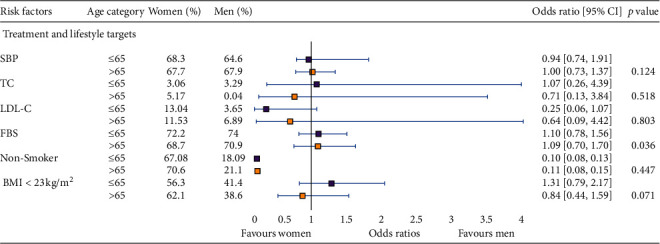
Age-adjusted sex differences in lifestyle factor management, stratified by age category.

Age-adjusted sex difference on risk factor management stratified by age category is presented in Figure 3. No significant difference was observed in achieving risk factor targets except for FBS (*p* < 0.05) where more men achieved control target compared with women in both age categories. In the less-than-65 category, 74% of men achieved control compared with 72.2% women with OR of 1.10 (95% CI 0.78 to 1.56), while in the more-tha-65 category, 70.9% men achieved control compared with 68.7% women with OR of 1.09 (95% CI 0.70 to 1.70). All other risk factor targets achieved a greater number of women compared with men; however, these differences were not significant (Figure 3).

Age=adjusted sex differences in lifestyle factor management, stratified by the age category. Odds ratios (95% CI) are presented as women versus men; *p* values are for interaction between subgroups. SBP, systolic blood pressure; TC, total cholesterol; LDL-C, low-density lipoprotein cholesterol; FBS, fasting blood sugar; BMI, body mass index.

## 4. Discussion

The present study among patients diagnosed with ACS across the island-wide network of hospitals in Sri Lanka highlighted the differences in risk factor prevalence and management between men and women. With regard to the prevalence of risk factors, hypertension, dyslipidemia, and diabetes were more prevalent among Sri Lankan women, while smoking was more in men. More sedentary lifestyle among Sri Lankan women and the genetic factors would have been contributed to this observation. Similar to findings from our study, the analysis of SURF study and PROMISE trials have showed higher rates of hypertension among women (M: 71.9% vs. F: 80.8% and M: 63.2% vs. F: 66.6%) [17, 18]. Furthermore, SURF study data have shown better blood pressure control among women, although this was not observed in our population. We also observed a higher prevalence of diabetes in women (M: 15.0% vs F: 19.7%). The PROMISE study showed a similar higher prevalence of diabetes in women, overall (M: 31.9% vs. F: 40.3%), and in subpopulations from Europe (M: 24.2% vs. F: 27.2%), Asia (M: 34.8% vs. F: 48.1%), and the Middle East (M: 71.6% vs. F: 86.7%) [7]. Interestingly, the prevalence of diabetes mellitus was much lower in our population compared with that of these studies [7, 18]. Diabetes causes myocardial ischemia by different mechanisms including atherosclerosis, microvascular dysfunctions, and coronary ion channel dysregulation due to oxidative stress. Coronary ion channels are important in the cross-talk between myocardial metabolism and coronary blood flow, and it may represent the link among coronary microvascular dysfunction, ischemic heart disease, and consequent heart failure. Specially, some genetic variants for ATP-dependent potassium channels seem to be involved in the determinism of IHD [19–21].

Similarly, much higher prevalence of dyslipidemia (without a significant sex difference) was observed in the PROMISE (M: 67.8% vs F: 67.1%) and SURF studies (M: 78% vs F: 73%). Smoking rates were significantly lower among women (1.4%) in our study population. The analysis of the SURF data showed lower rates of smoking among Asian women (2.7%) which agreed with our data. This is in contrast to those of the Western Population analyzed in SURF (11.0%) and PROMISE (14.4%) trials [7, 18]. Numerous studies, including systematic reviews, have shown that, for Asian women, the prevalence of smoking remains very low [22]. The observed differences in the smoking behavior of men and women are attributed to the differences in sociocultural environment and social circumstances in Asian populations [23].

In the Western population (e.g., SURF data), control of cardiovascular risk factors was generally poorer in women than in men. In contrast, Asian women are more likely than men to reach lifestyle targets [7]. A small-scale Indian study has shown overall poor control of risk factors in men compared with women [24]. However, data from the some of the Middle East countries including the southwest region of Saudi Arabia and Egypt have shown no sex difference in cardiovascular risk factor management [25, 26]. In a Korean population, no sex-based differences has been observed in ACS-related mortality and morbidity [27]. Similarly, in our population, except for LDL-cholesterol targets and FBS (only when stratified by age), no significant sex difference was observed in other risk factor target achievement between sexes. Higher literacy rates and widely available free health care facilities to the general public and sex equity in the Sri Lankan society may have contributed to timely seeking of medical care and better compliance with preventive strategies among women.

With regard to the use of preventive medications among ACS patients, our data show overall less than 30% of the patient using of aspirin, clopidogrel, *β*-blockers, ARB/ACE inhibitors, and statins as a primary preventive measure. There was a sex difference in the usage with significantly higher percentage of women using aspirin, statins, and ARB/ACE inhibitors. However, the use of same medicines as a secondary preventive measure exceeded 90% for antiplatelet medications and statins. Our data are almost compatible with SURF data in this regard [7, 13]. Compared with EUROASPIRE IV data, the use of ARB/ACE inhibitors as a secondary preventive measure is lesser in our population [13, 28]. This highlighted the delay in or not commencing ARB/ACE inhibitors before discharge which is an important issue to be addressed by the health care teams. Improved use of secondary preventive medication showed the strength of the public health sector in Sri Lanka where the medicines are given free of charge to all patients. This model is appropriate to be used in the resource poor settings in the region.

Regarding the assessment of GRACE risk scores, majority in our ACS patient population has fallen into high- and intermediate-risk categories. Young men seem to have higher risk compared with same-aged women which could be a result of hormonal protection in young women. Gulf RACE-2 data showed higher 1-month and 1-year mortality rates among women than men (11% vs. 7.4% and 17.3% vs. 11.4%, respectively, *p* < 0.001). It also showed both baseline and management differences contributed to a worse outcome in women [29]. Women sex was an independent predictor of in-hospital, 1-month, and 1-year mortality (only for STEMI), in Spain [30]. Women sex was an independent predictor of hospital mortality in the STEMI subpopulation in a ARIAM-SEMICYUC registry [31]. These results are in contact to the GRACE risk prediction in our population, where younger men (<65 years) were shown to have higher in-hospital and 1-year mortality. Risk stratification is a useful means of selecting patients in a resource poor setting. The GRACE score being a helpful guide for clinicians in non PCI-capable centers in early transfer to early intervention in STEMI patients after fibrinolytic therapy, we recommend risk assessment and documentation in ACS patients in all resource poor settings for resource prioritization [32].

Regarding the sex difference in the management of ACS, variable results have been observed. In the PROMISE trial, women were more often referred for imaging tests (adjusted odds ratio: 1.21; 95% confidence interval: 1.01 to 1.44) than nonimaging tests compared with men [18]. In a multivariate analysis of the MyRiAD data, women sex was associated with a lower referral for coronary angiography (HR 0.41, 95% CI 0.23–0.78, *p*=0.006) [33]. In the Gulf RACE-2 study, women underwent fewer invasive procedures including angiography (27.0% vs. 34.0%; *p* < 0.001), percutaneous coronary intervention (PCI) (10.5% vs. 15.6%; *p* < 0.001), and reperfusion therapy (6.9% vs. 20.2%; *p* < 0.001) than men [32]. Our ACSSLAP data also showed women receive lesser secondary prevention care compared with men, especially with regards to angiography and 2D-echocargiography. Therefore, sex difference when offering cardiac care and risk management needs to be minimised in any setting across the world. Overall, the rate of reperfusion therapy with PCI and CABG is low in Sri Lanka compared with that of the western countries. However, based on the initial results of this audit, the national guidelines were updated, and new facilities were allocated to regional centers. However, the situation needs further improvement.

ACSSLAP is the first island-wide audit in Sri Lanka and largest study in the South Asia population on acute coronary syndrome management. As MINAP audit standards have been followed, these data can be compared with global data filling an essential gap in the literature. However, there are limitations in the study such as omission of few hospitals due to lack of data, incomplete data due to incomplete investigations special serum lipid levels, and troponin level and data collection errors causing removal from the final data set. Incomplete data can affect the prevalence but may have not affected the data on management and sex difference. Treatment targets of risk factors vary widely across different populations. South Asian population lacks validated targets for risk factor control. Application of western population targets may have underestimated the control, and this necessitates the identification of own risk factor targets for our population. Currently, based on the audit results, several island-wide measures have been implemented. The second phase of the ACSSLAP is to be planned for removing deficiencies of the phase I.

## 5. Conclusion

Smoking is significantly lower among Sri Lankan women diagnosed with ACS. However, hypertension, diabetes, and dyslipidemia were more prevalent. There was no significant difference observed for most of the other risk factors and control targets. However, primary and secondary preventive strategies and management could be more improved in both sexes. ACSSLAP provides a model to other South Asian and low- to middle-income countries to conduct a comprehensive audit on the management of ACS. Furthermore, validated targets for risk factor control specific to South Asian population needed to be developed due to different genetic profiles, dietary habits and life-styles of this population.

## Figures and Tables

**Table 1:** Patient characteristics and cardiovascular risk factors and the management stratified by sex.

					Total	Men (%)	Women (%)	*p* value
Number					*n* = 2116	*n* = 1242 (58.7)	*n* = 874 (41.3)	<0.001
Age (years)					61.0 (12.6)	60.2 (12.3)	62.1 (12.9)	<0.001
Disease category								
STEMI					608 (28.7)	425 (34.2)	183 (20.9)	<0.001
NSTEMI					695 (32.8)	427 (34.4)	268 (30.7)	0.073
UA					813 (38.4)	390 (31.4)	423 (48.4)	<0.001
Smoking status								
Current smoker					327 (15.4)	315 (25.4)	12 (1.4)	<0.001
Ex-smoker					348 (16.4)	334 (26.9)	14 (1.6)	<0.001
Passive smoker					75 (3.5)	12 (0.9)	63 (7.2)	<0.001
Never smoker					864 (40.8)	243 (19.6)	621 (71.1)	<0.001
Known medical history								
Hypertension					994 (45.6)	492 (39.6)	502 (57.4)	<0.001
Dyslipidemia					358 (16.4)	186 (15.0)	172 (19.7)	0.004
CHD					370 (17.0)	205 (16.5)	165 (18.9)	0.157
Diabetes					615 (28.2)	319 (25.6)	296 (33.9)	<0.001
Physical and laboratory measurements								
BMI (kg/m^2^)					23.4 (4.0)	23.3 (3.6)	23.4 (4.6)	0.93
SBP (mmHg)					136.7 (29.2)	136.8 (28.8)	136.5 (29.7)	0.815
TC (mmol/L)					199.8 (90.4)	205.1 (109.5)	191.2 (42.4)	0.129
LDL-C (mmol/L)					123.0 (38.3)	129.1 (38.1)	113.6 (37.1)	0.008
FBS (mmol/L)					120.7 (58.9)	119.5 (53.2)	122.4 (66.0)	0.421
Haemoglobin (g/dL)					12.2 (1.9)	12.6 (2.0)	11.5 (1.6)	<0.001
Serum creatinine (mg/dL)					1.17 (0.6)	1.22 (0.6)	1.07 (0.6)	<0.001
Medications (on admission)								
Aspirin					441 (20.8)	236 (19.0)	205 (23.4)	0.013
Clopidogrel					332 (15.7)	183 (14.7)	149 (17.0)	0.150
*β*-Blocker					222 (10.5)	120 (9.7)	102 (11.7)	0.138
ARB/ACE inhibitor					539 (25.4)	269 (21.6)	270 (30.9)	<0.001
Stain					552 (26.1)	293 (23.6)	259 (29.6)	0.002
Medications (secondary prevention)								
Aspirin					2065 (97.6)	1216 (97.9)	852 (97.5)	0.519
Clopidogrel					2065 (97.6)	1218 (98.1)	851 (97.4)	0.283
*β*-Blocker					616 (29.1)	370 (29.8)	246 (28.1)	0.412
ARB/ACE inhibitor					1162 (54.9)	671 (54.0)	491 (56.2)	0.327
Stain					2081 (98.3)	1230 (99.0)	861 (98.5)	0.275
Management								
Reperfusion attempted					437 (20.6)	257 (20.7)	180 (20.6)	0.957
Dietary advices given					1650 (78.0)	946 (76.2)	704 (80.5)	0.017
Cardiac rehabilitation started					759 (35.9)	453 (36.5)	306 (35.0)	0.519
Angiogram done/planned					239 (11.3)	160 (12.9)	79 (9.0)	0.006
CABG done/planned					52 (2.4)	37 (3.0)	15 (1.7)	0.088
2D-Echo done/planned					1308 (61.8)	804 (64.7)	504 (57.7)	0.001
TMT done/planned					461 (21.8)	265 (21.3)	196 (22.4)	0.550
Cardiology referral done/planned					1281 (60.5)	758 (61.0)	532 (60.9)	0.940

Results are shown as mean (SD) for continuous variables and percentage (number of observations/nonmissing data) for categorical variables. STEMI, ST elevation myocardial infarction; NSTEMI, non-ST elevation myocardial infarction; UA, unstable Angina; CHD, coronary heart disease; BMI, body mass index; SBP, systolic blood pressure; TC, total cholesterol; LDL-C, low-density lipoprotein cholesterol; FBS, fasting blood sugar; ARB/ACEI, angiotensin II receptor blocker/angiotensin-converting enzyme inhibitor: CABG, coronary artery bypass grafting; TMT, treadmill stress testing.

**Table 2:** GRACE Risk assessment of post-ACS patients stratified by sex and the risk level.

GRACE risk assessment	Category	Total *n* = 2116	Men *n* = 1242	Women *n* = 874	*p* value
In-hospital mean mortality risk	≤65 years	1.6 (2.1)	1.7 (2.3)	1.4 (1.9)	0.056
	>65 years	4.3 (5.2)	4.4 (5.4)	4.3 (4.9)	0.798

6-month post-discharge mean mortality risk	≤65 years	3.6 (4.2)	3.7 (3.2)	3.5 (5.3)	0.447
	>65 years	10.7 (8.9)	10.7 (8.9)	10.8 (9.0)	0.846

1-year post-discharge mortality risk	≤65 years	4.2 (3.7)	4.2 (4.0)	3.9 (3.2)	0.027
	>65 years	12.0 (10.4)	12.3 (10.8)	11.7 (9.9)	0.451

1-year post-discharge mortality risk category	Low	273 (13.9)	163 (59.7)	110 (40.3)	0.555
	Intermediate	836 (42.8)	472 (56.4)	364 (43.6)	0.211
	High	842 (43.1)	498 (59.1)	344 (40.9)	0.403

3-year post-discharge mortality risk	≤65 years	10.5 (9.5)	10.7 (9.9)	10.5 (8.8)	0.861
	>65 years	27.6 (18.3)	27.1 (18.1)	28.3 (18.6)	0.409

3-year post-discharge mortality risk category	Low	309 (15.8)	167 (54.0)	142 (46.0)	0.054
	Intermediate	709 (36.3)	418 (58.9)	291 (41.1)	0.979
	High	933 (47.7)	566 (60.6)	367 (39.4)	0.151

GRACE risk scores between men and women are shown stratified by age group or the risk level shown at different timelines for acute coronary syndrome patients and the differences are calculated. GRACE score, the Global Registry of Acute Coronary Events score.

## Data Availability

The data used to support the findings of this study will be made available from the corresponding author upon request.
